# Ultrasonographic Ventral Hip Joint Approach and Relationship with Joint Laxity in Estrela Mountain Dogs

**DOI:** 10.3390/ani15040547

**Published:** 2025-02-13

**Authors:** Inês Tomé, Sofia Alves-Pimenta, Bruno Colaço, Mário Ginja

**Affiliations:** 1 Department of Veterinary Sciences, University of Trás-os-Montes e Alto Douro, 5000-801 Vila Real, Portugal; inestome@utad.pt; 2 CECAV, Centre for Animal Sciences and Veterinary Studies, University of Trás-os-Montes e Alto Douro, 5000-801 Vila Real, Portugal; salves@utad.pt (S.A.-P.); bcolaco@utad.pt (B.C.); 3 Department of Animal Science, University of Trás-os-Montes e Alto Douro, 5000-801 Vila Real, Portugal

**Keywords:** hip dysplasia, joint laxity, ultrasound, cranial recess, dogs

## Abstract

Hip dysplasia (HD) is a prevalent disease in medium- to large-breed dogs, characterized by joint laxity and degenerative joint changes. The early diagnosis of HD poses significant challenges, as radiographic imaging often identifies the disease only in advanced stages. Conversely, ultrasonography, a non-invasive and cost-effective imaging modality, offers the potential for earlier detection by evaluating the surrounding soft tissues and synovial changes. This study aimed to assess the relationship between the ventral hip ultrasonographic findings, and hip joint laxity evaluated through stress radiographs on 22 young Estrela Mountain dogs (*n* = 44 hips) aged 4 to 8 months. Key ultrasound measurements included synovial fluid in the cranial femoral neck recess (CFNR) and capsular-synovial fold thickness (CFT). Radiographic laxity was estimated by measuring the distraction index (DI). The median (quartile 25–75%) of the CFNR area, CFT, and DI were 44.00 (27.00–52.25) mm^2^, 3.10 (2.68–3.55) mm, and 0.38 (0.34–0.40), respectively. The Spearman correlation coefficient was statistically significant between all of these variables (*p* < 0.05). The ventral ultrasonographic approach to the hip joint revealed potential, considering the early diagnosis of HD in dogs, by showing relationships between changes in periarticular soft tissues and joint laxity. Further studies are needed to associate ultrasonographic findings with radiographic signs of HD and related clinical signs in dogs.

## Simple Summary

Hip dysplasia (HD) is an orthopedic disease that affects large-sized dogs, causing pain and damage to the hip joint. Diagnosing HD is challenging because X-rays often detect this condition in late stages. Our study explored the use of ultrasound, a safer and non-invasive diagnostic tool, to identify early signs of HD progression in Estrela Mountain dogs. Using a ventral approach to the hip joint, we evaluated a series of measurements and correlated them with the distraction index, an X-ray measurement that evaluates joint looseness. We found a strong correlation between these measurements, showing that the ultrasound can detect early hip changes, associated with late HD development. Our findings can help veterinarians identify hip dysplasia earlier, leading to better treatment options and an improved quality of life in dogs.

## 1. Introduction

**Figure 1:**
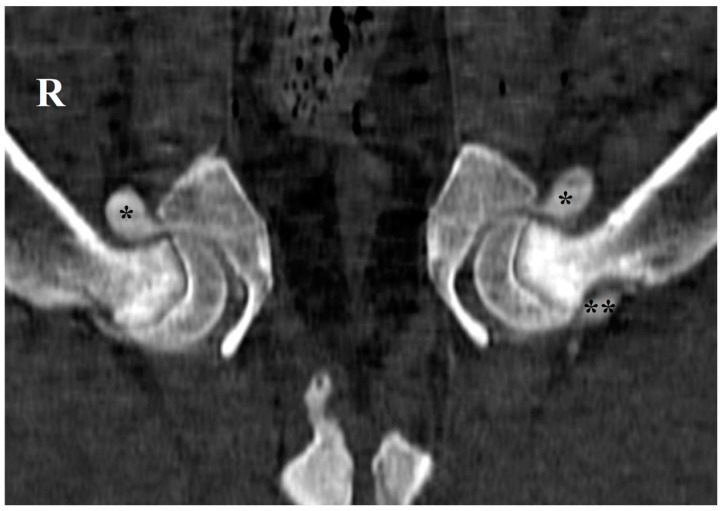
Computed tomography image in the dorsal plane of the hip joint of an Estrela Mountain dog cadaver, 4 months old, in dorsal recumbency and with the hindlimbs abducted, after intraarticular contrast medium administration, highlighting the cranial (*) and caudal (**) femoral neck recesses. R: right side.

The complex mobility of the canine hip joint relies on the coordinated movement of several anatomical structures, including synovial fluid, cartilage, bones, ligaments, tendons, blood vessels, nerves, and muscles [1,2]. The hip joint is characterized by the existence of two articular recesses, one cranial and the other caudal to the femoral neck, where synovial fluid preferentially accumulates [3] (Figure 1). The synovial fluid, accumulating in the cranial and caudal femoral recesses, acts as an important lubricant and shock absorber, reducing friction during movement and protecting the joint [3,4,5]. Maintaining the integrity of these structures is essential for a proper and healthy hip function [1,2].

Hip dysplasia (HD) is one of the most common hip disorders affecting medium- and large-breed dogs [6,7,8,9,10]. It is characterized by degenerative joint disease, resulting in bone remodeling of the acetabulum and the femoral head [9,11,12]. The diagnosis of HD is usually made by using a standard ventrodorsal radiograph taken under sedation or general anesthesia. Moreover, the earliest detectable radiographic sign of HD is hip joint laxity, which is considered the major risk factor for the development and progression of HD [5,13]. Hip joint laxity is assessed using the distraction index (DI), a radiographic parameter that quantifies the extent of the femoral head displacement relative to the acetabulum, in a ventrodorsal hip stress radiograph obtained with a hip distractor [5,14,15]. Furthermore, early bone changes characteristic of HD, are preceded by abnormalities in synovial fluid composition and the surrounding joint soft tissues, which result in increased joint laxity [3,5,15]. These early joint changes are especially observed in young animals that are not yet exhibiting clinical signs [3,13]. Increment in the hip synovial fluid volume was associated with higher joint laxity in dogs [16].

Ultrasonography is a widely used and cost-effective imaging modality in veterinary medicine, particularly for the diagnosis of musculoskeletal conditions in small animal patients, due to its capacity to identify soft tissue injuries and superficial bone changes [17,18]. Unlike radiography, ultrasonography offers the advantage of dynamic imaging, allowing real-time visualization of joint structures, synovial fluid, and soft tissue interactions [17]. Moreover, it does not expose patients or the operator to ionizing radiation, offering a safer imaging modality [19,20]. This imaging technique can be performed in awake dogs with minimal physical restraint, requiring sedation or general anesthesia only in exceptional cases [17]. Hip ultrasound can be performed using dorsal or ventral approaches, with the ventral approach offering the advantage of not requiring trichotomy due to reduced hair density in the region and resulting in less aesthetic impact [17,18,20,21].

Previous studies have demonstrated the utility of ultrasonography in evaluating musculoskeletal injuries involving the shoulder, elbow, supraspinatus and biceps tendon, and iliopsoas, and for guided hip administrations [18,22,23,24,25]. However, literature on hip ultrasonography in dogs remains scarce [20]; additional studies are needed to investigate normal hip sonoanatomy and assess its utility in diagnosing hindlimb pathologies potentially associated with the hip joint.

This study aimed to evaluate the variability in ultrasonographic imaging of hip bone structures and surrounding soft tissues using the ventral approach in young Estrela Mountain dogs, a breed with a high prevalence of HD [6,7,8]. Another objective of this study was to find a relationship between the ultrasonographic and DI measurements. This research will be important to determine whether ultrasonography can reliably detect early abnormalities, such as synovial thickening or increased synovial fluid volume, which may be associated with hip laxity, HD, or related disorders. We hypothesized that hip ultrasonography would effectively identify some early soft tissue changes. In contrast, late-stage osteoarthritic changes, namely changes in the cortical echogenicity of the femoral neck and periosteum, femoral head subchondral bone, and articular cartilage, would not yet be detectable. For this purpose, special attention was given to these hip joint structures during the acquisition and analysis of ultrasound images in young animals.

## 2. Materials and Methods

### 2.1. Animals

Dogs presenting for screening HD at the Veterinary Hospital of the University of Trás-os-Montes and Alto Douro (UTAD) in 2024 were prospectively enrolled in this diagnostic accuracy study with informed owner consent. Inclusion criteria included animals aged four to twelve months from the Estrela Mountain dog breed. Exclusion parameters were prior trauma or surgery to the hip joint or pelvic limb and a poor ultrasound quality of the images recorded, which does not allow for a proper assessment of the hip joint. A total of 22 dogs (44 hips) were included in this study. Ultrasound studies were performed and analyzed by IT and radiographic views were performed and analyzed by MG.

### 2.2. Radiographic Hip Stress View and Hip Laxity Measurement

The radiographic image acquisition was performed with the dogs under deep sedation, using butorphanol (Butomidor^®^, Richter Pharma AG, Wels, Austria, at 0.2 mg/kg) and dexmedetomidine (Sedadex^®^, Le Vet Beheer B.V., Utrecht, The Netherlands, at 4 μg/kg) intramuscularly, and propofol (Propofol Lipuro^®^, B.Braun, Lisbon, Portugal, at 4 mg/kg) intravenously. The sedation was reversed with atipamezole hydrochloride (Antisedan^®^, Orion Corporation, Espoo, Finland, IM).

**Figure 2:**
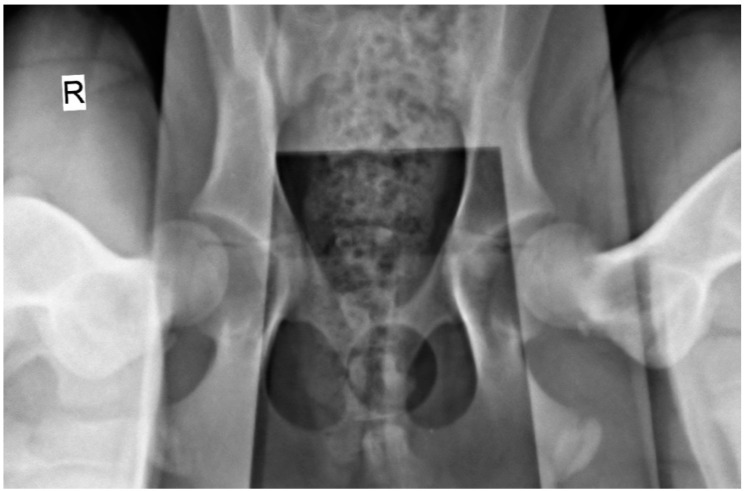
Distraction radiographic view obtained with the hip distractor DisUTAD of a male dog with 4 months of age and a body weight of 20 kg. The image shows great joint laxity bilaterally, evidenced by the separation between the femoral head and the acetabulum. A distraction index of 0.65 and 0.62 was registered in the right and left joints, respectively. R: right side.

The radiographic stress views were obtained with dogs placed in the supine position on the X-ray table (Optimus 80, Philips, Amsterdam, The Netherlands), with both femurs in a neutral position and the distractor device (DisUTAD, University of Trás-os-Montes and Alto Douro, Vila Real, Portugal) placed symmetrically between them. Then, the examiner raised and adducted the femurs against the distractor and the stress hip projection was obtained (Figure 2).

The hip laxity measurements were performed in a semiautomatic software (Horos, version 4.0.0 RC5, New York, USA). The hip laxity was measured by calculating the DI. First, the femoral head and acetabulum were both delimitated by a circumference, and the distance between both circumference’s centers was calculated and then divided by the radius of the femoral head [5].

### 2.3. Ultrasonographic Ventral Hip Joint Approach

All ultrasound examinations were performed using a portable ultrasound machine (Logiq e, General Electric Medical Systems, Buc, France) with a high-frequency linear probe (L8-18iRS, General Electric Medical Systems, Buc, France), configured with a musculoskeletal preset, at a frequency of 16 MHz. Dogs were positioned in the supine position with the hindlimb flexed 90° and abducted using a ventral approach to the hip joint. Hair clipping was not performed, and acoustic gel was applied to the skin to ensure an adequate acoustic coupling of the transducer. To minimize variability in the image acquisition, a standardized protocol for probe positioning and image acquisition was established as follows:

- Longitudinal Femoral Head–Neck Plane

**Figure 3:**
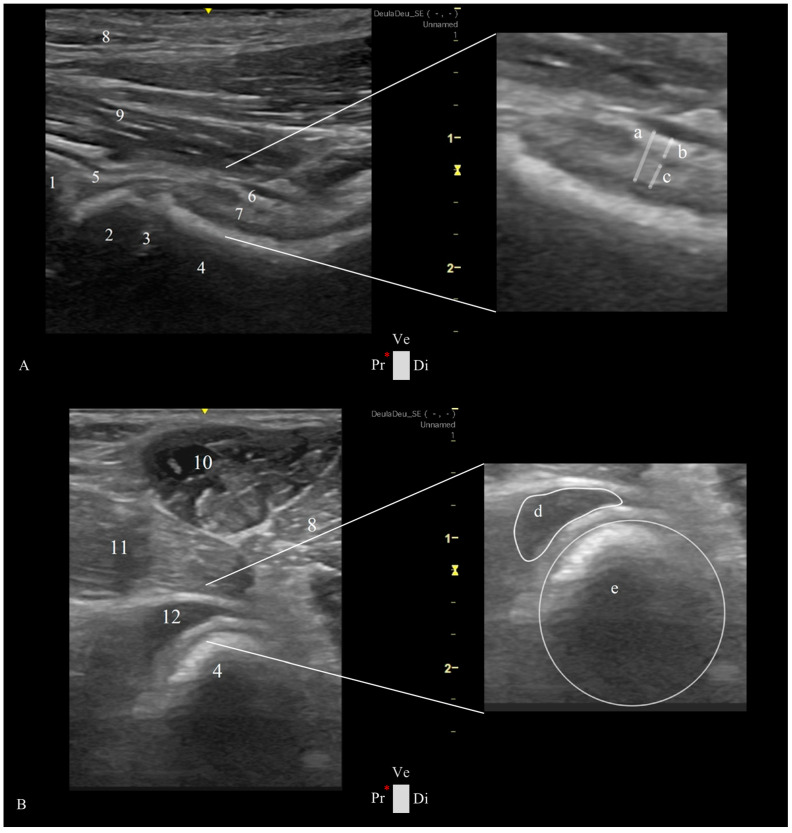
Ultrasound views were obtained in a ventral approach to the right hip joint in a female dog at 4 months of age and weighing 20 kg. (**A**) Longitudinal plane of the femoral head–neck, showing on the right side a magnified view of the joint capsule with measurements performed in the study: (a) capsular-synovial fold thickness and (b) outer and (c) inner synovial membrane thicknesses of 2.30, 0.80, and 1.00 mm, respectively. (**B**) Transverse plane of the femoral head–neck showing on the right side a magnified view of the transverse plane of the cranial femoral neck recess and femoral neck, with measurements performed in the study: (d) white line delimitating the anechoic signal of the synovial fluid within the cranial femoral neck recess of 25.00 mm^2^ and (e), circumference delimitating the femoral neck with a diameter of 16.00 mm. (1) ventral acetabular rim, (2) femoral head, (3) proximal femoral growth plate, (4) femoral neck, (5) joint capsule, (6) outer synovial membrane layer, (7) outer synovial internal synovial lining, (8) adductor magnus et brevis muscle, (9) adductor longus muscle, (10) pectineus muscle, (11) iliopsoas muscle, and (12) transverse view of the cranial femoral neck recess. Ca: caudal, Cr: cranial, Di: distal, Pr: proximal, Ve: ventral, and *: probe orientation and indication marker. The yellow scale represents the depth of the ultrasound image in centimeters and the focus zone (⧖).

The transducer was placed over the femoral neck and head, directed distoproximally, caudal to the pectineus muscle, with the indicator marker oriented toward the proximal edge of the femur and represented by the left side of the screen. The longitudinal femoral head–neck plane showed three longitudinal muscle layers of adductor magnus et brevis, adductor longus, and iliopsoas; a transverse view of the deep branch of the medial circumflex femoral artery and vein, the hip joint capsule, articular space, and synovial lining; the acetabulum; and the femoral head (Figure 3A).

- Transverse Femoral Head–Neck Plane

The probe was placed over the pectineus muscle and transverse to the femoral diaphysis, with the indication marker oriented cranially, and then moved distoproximally. Upon reaching the hip joint, the transducer was tilted cranially until allowing the visualization of the acetabulum, femoral head, and neck. Sliding the transducer from the cranial to the caudal ventral acetabular rim provided a comprehensive view of the femoral head–neck and a transverse plane of the cranial femoral neck recess (CFNR). The pectineus, adductors, and iliopsoas were some of the muscles observed in this plane (Figure 3B).

### 2.4. Ultrasonographic Hip Joint Measurements

The longitudinal femoral head–neck plane included three measurements at the level of the femoral neck: capsular-synovial fold thickness (CFT), a perpendicular distance between the external limit of the joint capsule and inner synovial membrane layer, and the outer and inner synovial membrane thickness, a perpendicular distance between their delimitation (Figure 3A). In the transverse femoral head–neck plane was performed two measurements: the femoral neck diameter, by drawing a circumference that encompasses the circular cortical bone echogenicity, and the CFNR area, by drawing a free line over the external limit of the anechoic signal of the synovial fluid (Figure 3B). To reduce the influence of the dog’s size variability, a CFNR index was created, and it is calculated by dividing the CFNR area by the femoral neck diameter. All images were analyzed using a free DICOM Medical Image Viewer Software (Horos, version 4.0.0 RC5).

### 2.5. Statistical Analysis

Statistical analysis was performed using commercially available software (SPSS Statistics for Windows, Version 27.0, IBM, New York, NY, USA). Data were tested for normality using the Shapiro–Wilk test. Non-parametric variables were analyzed using Spearman’s correlation analysis (ρ) to assess the relationship between ultrasonographic and radiographic variables [26]. The basic features of the data were presented using minimum, maximum, and mean ± standard deviation (SD) or median (quartile 25–75%) for normal and non-normal variables distribution, respectively [26]. A *p* value of <0.05 was considered statistically significant.

## 3. Results

The study evaluated 22 Estrela Mountain breed dogs (*n* = 44 hips), 11 males and 11 females, aged from 4 to 8 months with a mean ± SD of 4.73 ± 1.26 months, with a body weight of 22.73 ± 6.80 kg. The minimum, maximum, and mean ± SD of the ultrasonographic and radiographic measurements obtained in the study (CFT, outer and inner synovial membrane thickness, femoral neck diameter, CFNR area, CFNR index, and DI) are summarized in Table 1.

In all animals, the cortical bone of the periarticular structures as well as the subchondral bone of the femoral head revealed linear and regular echogenicity, not evidencing changes compatible with the development of osteophytes or other signs of degenerative joint disease.

**Figure 4:**
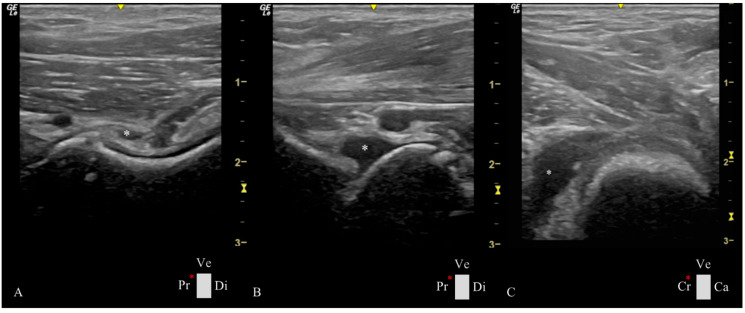
Ultrasonographic images of Estrela Mountain dog hips at 4 months of age present a considerable amount of synovial fluid (white *) in different compartments of the hip joint. The hip joints have a great joint laxity with a distraction index of 0.60. (**A**) Longitudinal plane of the femoral head–neck plane showing synovial fluid between the outer and inner synovial membrane and an increased CFT of 3.40 mm. (**B**) Longitudinal plane of the femoral head–neck in which fluid accumulation was observed in the ventral aspect of the hip joint. (**C**) Transverse plane of the femoral head–neck displaying a cranial femoral neck recess with an area of 79.00 mm^2^. Ca: caudal, Cr: cranial, Di: distal, Pr: proximal, Ve: ventral, and red *: probe orientation and indication marker. The yellow scale represents the depth of the ultrasound image in centimeters and the focus zone (⧖).

The Spearman correlation was statistically significant and positive among many of these variables (Table 2). We highlight the strong correlation between the evidence of joint fluid in the ultrasound image CFNR area (ρ = 0.81; *p* < 0.01) and CFNR index (ρ = 0.85; *p* < 0.01) with DI (joint laxity) obtained in stress radiographs. In animals with a higher DI, synovial fluid is evident in different joint spaces (Figure 4).

## 4. Discussion

Plain radiographs have been used for decades as the main reference for hip osteoarthritis diagnosis [12], despite ultrasonography gaining popularity in the last few years in the musculoskeletal field [19]. Currently, ultrasound is equipped with high-frequency transducers which have facilitated its widespread adoption in veterinary medicine, offering an affordable tool that provides an excellent image quality of superficial musculoskeletal structures [20,27]. Our study has demonstrated the utility of ultrasonography for detecting soft tissue changes in young Estrela Mountain breed dogs, associated with hip laxity.

Our initial research hypothesis was confirmed as hip ultrasonography effectively identified soft tissue changes, including CFT and joint synovial fluid area, which showed a statistically significant association with hip laxity evaluated in stress radiographs. Conversely, late-stage osteoarthritic bone changes linked to HD were absent in both ultrasound and radiographic evaluations. These findings are in agreement with previous research that underscores soft tissue changes linked to HD. For instance, Ginja et al. (2009) [3] and Smith et al. (1990) [5] identified synovial thickening and increased synovial fluid volume as critical indicators of joint laxity and HD. Similarly, Sudula (2016) [19] and Ginja et al. (2009) [3] emphasized the relevance of recess area measurements, which were found to be correlated with synovial effusion and capsular thickness.

Osteoarthritic changes on the hip, characterized by periarticular bone irregularities observable through the ultrasound, tend to manifest later, following soft tissue changes and joint instability [2,3,5,27,28]. In our study, the ventral ultrasonographic hip approach provided a clearer and more standardized imaging of the CFNR compared to the caudal recess. The difference is likely due to anatomical positioning relative to the femoral neck and varying muscular coverage. For this reason, the caudal recess evaluation was not included in this study. Additionally, in the longitudinal view of the cranial recess, identifying an alternative anatomical landmark to the femoral neck for image referencing proved challenging. Therefore, we used the transverse CFNR plane, where the circular echogenicity of the femoral neck cortical bone consistently appeared caudally, serving as a reliable reference point. When assessing the outer and inner synovial membrane, we consistently aimed to capture images on the central plane of the femoral neck to exclude recesses. Some statistically significant correlations were observed, such as those between the outer and inner synovial membrane thickness and CFT or between the CFNR area and CFNR index, which may result from a direct relationship/dependence between these variables and may not hold substantial clinical significance. The significant correlations between age and the different ultrasonographic measurements, namely CFT, outer and inner synovial membrane thickness, synovial fluid area, and femoral neck diameter were anticipated and may reflect the different growth stages of the animals. Notably, the age-related correlations decreased when indices like the CFNR index and DI were applied, which is consistent with similar studies [3,5]. Thus, normalizing the CFNR area by dividing it with the femoral neck diameter for the determination of the CRFN index, mitigated the influence of the dogs’ size, as evidenced by a stronger correlation between CRFN index and hip laxity (ρ = 0.85). These findings emphasize the efficacy of ultrasonography in the early detection of soft tissue changes associated with HD, offering a non-invasive and reliable diagnostic tool that complements traditional radiographic imaging techniques.

The ventral ultrasound approach to the hip joint described in this study holds significant clinical potential for the early detection of HD. One major challenge in this anatomical region is that HD often goes unnoticed in its early developmental stages. Consequently, definitive radiographic diagnosis tends to occur later, primarily due to clinical or reproductive purposes (e.g., selecting breeding), emphasizing the need for an early, straightforward, cost-effective imaging method that seamlessly integrates preventive care into the veterinary routine practice. The methodology presented in this research addresses these needs by avoiding deep sedation or general anesthesia, reducing its costs; avoiding the need for trichotomy by adopting the ventral approach to the hip joint, being less time-consuming; and, also, eliminating the exposure to ionizing radiation, diminishing the harmful effects to the public health.

Significant variability was observed in the CFNR index, ranging from 0.88 to 6.43 mm². This variability may reflect individual differences in synovial fluid dynamics, joint structures, or fluid accumulation associated with different joint movements during imaging examination. Understanding these variations is critical for refining ultrasonographic diagnostic criteria for HD and related disorders. These findings reinforce the predictive value of ultrasonography in assessing early-HD-related changes. Additionally, our observations regarding femoral neck diameter variability mirror the findings of Weigel and Wasserman (1992) [1], who associated such changes with altered biomechanical stress in the hip joint. The study by Greshake and Ackerman (1993) [21] further supports the notion that subtle differences in the femoral neck reflect underlying joint instability. These comparisons highlight the clinical utility of ultrasonographic parameters as early indicators of HD, providing a solid foundation for incorporating such imaging tool into veterinary practice. Nonetheless, our study includes limitations related to experienced operator dependency, challenges in standardizing measurements, or animal anatomical particularities, such as obesity, which may have caused a poorer ultrasound image quality. Future studies should correlate ultrasonography findings with clinical outcomes, tracking the progression over time of these findings and correlating them with radiographic parameters to further validate their utility and diagnosis capacity.

## 5. Conclusions

This study highlights the utility of ultrasonography as a non-invasive, cost-effective tool for the early detection of HD-related changes in young Estrela Mountain Dogs. By identifying early soft tissue markers associated with joint laxity, the ventral approach to the hip joint could enhance early detection and help define intervention strategies that will improve canine health and benefit breeding programs or HD screening. Further studies with larger sample sizes, other breeds, and longitudinal designs are essential to validate the clinical application of ultrasonography and refine predictive methodologies.

## Figures and Tables

**Table 1:** Descriptive statistics of the ultrasonographic and radiographic measurements in 22 (44 hips) Estrela Mountain breed dogs.

			N	Minimum	Maximum	Mean ± SD or Median (Quartile 25–75%)
Ultrasonographic Measurements	Longitudinal View	Capsular-Synovial Fold Thickness *	44	1.7 mm	6.31 mm	3.10 (2.68–3.55) mm
		Outer Synovial Membrane Thickness	44	0.50 mm	2.50 mm	1.37 ± 0.42 mm
		Inner Synovial Membrane Thickness *	44	0.70 mm	2.50 mm	1.20 (1.00–1.33) mm
	Transverse View	Femoral Neck Diameter *	44	13.00 mm	22.00 mm	17.00 (15.98–18.00) mm
		CFNR Area *	44	15.00 mm^2^	135.00 mm^2^	44.00 (27.00–52.25) mm^2^
		CFNR Index *	44	0.88	6.43	2.42 (1.59–3.13)
Radiographic Measurements		Distraction Index *	44	0.20	0.65	0.38 (0.34–0.40)

* Variable with non-normal distribution in Shapiro–Wilk test; CFNR: cranial femoral neck recess; N: number of hips.

**Table 2:** Spearman correlations and statistical significance among ultrasonographic and radio-graphic measurements in 22 (44 hips) Estrela Mountain breed dogs aged from 4 to 8 months.

Variables in the Study	Ultrasonographic Measurements						Radiographic Measurements
	Longitudinal Femoral Head–Neck Plane			Transverse Femoral Head–Neck Plane			
	Capsular-Synovial Fold Thickness	Outer Synovial Membrane Thickness	Internal Synovial Lining Thickness	Femoral Neck Diameter	CFNR Area	CFNR Index	Distraction Index
Capsular-Synovial Fold Thickness							0.55 *
Outer Synovial Membrane Thickness	0.85 *						0.52 *
Inner Synovial Membrane Thickness	0.50 *	0.70 *					0.30 *
Femoral Neck Diameter	0.00	−0.03	0.14				−0.12
CFNR Area	0.71 *	0.58 *	0.38 *	0.09			0.81 *
CFNR Index	0.66 *	0.63 *	0.35 *	−0.17	0.96 *		0.85 *
Age	0.19	0.23	0.36 *	0.52 *	0.19	0.05	0.11

CFNR: cranial femoral neck recess. The superscript * represents a statistically significant correlation between the variables.

## Data Availability

The original contributions presented in the study are included in the article, further inquiries can be directed to the corresponding author.

## Abbreviations

The following abbreviations are used in this manuscript:

HD	Hip Dysplasia
CFNR	Cranial Femoral Neck Recess
CFT	Capsular-Synovial Fold Thickness
DI	Distraction Index
SD	Standard Deviation
ρ	Spearman Correlation
